# Real-Life Data of Tirzepatide Use to Support Lifestyle Modification in Patients with Metabolic Syndrome

**DOI:** 10.3390/nu18081275

**Published:** 2026-04-17

**Authors:** Joanna Śledziona, Wojciech Warchoł, Marcin Mardas, Bogna Grygiel-Górniak, Michał Nowicki, Radosław Osmański, Marta Stelmach-Mardas

**Affiliations:** 1 Department of Medical Education and Communication, Poznan University of Medical Sciences, Rokietnicka Str. 7, 60-806 Poznan, Poland; fitlinefood@gmail.com (J.Ś.); mnowicki@ump.edu.pl (M.N.); 2 Department of Optometry, Poznan University of Medical Sciences, Rokietnicka Str. 5D, 60-806 Poznan, Poland; wwarchol@ump.edu.pl; 3 Department of Gynecological Oncology, Poznan University of Medical Sciences, Szamarzewskiego Str. 84/82, 60-101 Poznan, Poland; marcin.mardas@skpp.edu.pl; 4 Department of Rheumatology, Rehabilitation and Internal Diseases, Poznan University of Medical Sciences, 28 Czerwca 1956 Str. 135/147, 61-545 Poznan, Poland; bgrygiel@ump.edu.pl; 5 Department of Oncology and Immuno-Oncology, Greater Poland Cancer Centre, Garbary Str. 15, 61-866 Poznan, Poland; radoslaw.osmanski@wco.pl

**Keywords:** obesity, tirzepatide, nutrition, quality of life

## Abstract

**Background**: Tirzepatide is a novel therapeutic option for the management of metabolic disorders which has started to be implemented in routine practice. The study aimed to analyze the effectiveness of tirzepatide use and patient education in the field of healthy eating and weight loss, based on real-life data from the practice of a primary care physician, in metabolic syndrome (MetSyn) patients during a one-year follow-up period. **Methods**: This is a retrospective study based on real-life data of 118 MetSyn patients who were under the supervision of a general practitioner (GP). Analysis was conducted on 62 patients supported by trizepatide (2.5 mg for 4 weeks, then 5 mg for 4 weeks and 7 mg for 46 weeks) with dietary education and 56 patients that underwent dietary education with motivation only. Lipid profile, glucose level and blood pressure were assessed. Body Mass Index (BMI), waist-to-height ratio (WHtR), A Body Shape Index (ABSI), Lipid Accumulation Product (LAP), Visceral Adiposity Index (VAI) and Body Roundness Index (BRI) were calculated. The KomPAN^®^ questionnaire was used for dietary assessment and WHO Quality of Life-BREF for the quality of life assessment at 52 weeks. **Results**: Patients from both groups significantly reduced their body weight and WC and the values of the following indices: BMI, WHtR, ABSI, LAP and BRI. A significant increase in LDL cholesterol and triglyceride values was observed in both groups and a significant decrease in glucose level only in the group with tirzepatide combined with dietary modification. Energy value, energy density of food and nutrient intake did not differ between groups, while the intensity of beneficial nutritional features (pHDI-10) was low. Significant differences in patients’ QoL were observed, especially in the domain related to mental health (higher in trizepatide + diet group). **Conclusions**: Support in primary care by a physician was successful from a long-term perspective in the group using tirzepatide in combination with diet modification as well as in the group based on dietary modification only. The data do not indicate a significant advantage of any one approach for patients, prioritizing an individualized approach to treatment.

## 1. Introduction

The prevalence of people with obesity and metabolic syndrome (MetSyn) is increasing, which is related to a number of factors, including the development of negative eating patterns (high-fat and high-sugar diet and alcohol consumption), low physical activity, and stress [1]. MetSyn diagnostic criteria emphasize multimorbidity and the need to work with the patient at the primary care level, both using motivational training in the field of diet and weight reduction and supporting it with modern pharmacotherapy [1,2,3]. Recent years have brought about a number of effective methods of combating obesity—from advanced surgical methods to the use of drugs originally used in the treatment of diabetes and in weight loss, which are now effectively used in everyday medical practice [4,5,6,7].

Recently, the attention of clinicians has been mostly drawn to the use of GLP-1 analogs such as semaglutide and tirzepatide. Tirzepatide, as a dual long-acting glucose-dependent insulinotropic polypeptide receptor and glucagon-like peptide-1 receptor agonist, has direct effects on insulin secretion and sensitivity, appetite and metabolism [8]. The dual GLP-1R and GIPR agonism of tirzepatide is a novel therapeutic option for the management of metabolic disorders [8]. Importantly, tirzepatide has been available in Poland since 2024, but it is not reimbursed and its availability can be limited. For this reason, real-life data obtained from the routine work of primary care physicians are becoming increasingly valuable; a physician is the first point of contact and can implement treatment for people with MetSyn, adapting it to the health condition and capabilities of the patient. There is still the issue of effective dietary support combined with motivational training conducted during a GP visit, which allows for real changes in the eating habits of people with obesity [9,10]. Success in reducing the body weight of patients with obesity translates into their daily functioning in various areas of life, significantly improving quality of life (QoL) and reducing healthcare costs for the treatment of obesity complications [11].

The aim of the study was to analyze the effectiveness of tirzepatide use and patient education in the field of healthy eating and weight loss, based on real-life data from the practice of a primary care physician, in patients suffering from metabolic syndrome during a one-year follow-up period.

## 2. Materials and Methods

### 2.1. Patients’ Characteristics

The study protocol was approved by the Bioethical Committee at Poznan University of Medical Sciences (KB-652/23). The study was carried out in accordance with the Declaration of Helsinki. Only adults with complete clinical and selected biochemical data that had completed the selected questionnaires were analyzed. The results on biochemistry, anthropometry and blood pressure were analyzed at the beginning of education and after one year, while the data from the quality of life (QoL) questionnaire and the assessment of the frequency of food consumption (FFQ) were obtained after one year of lifestyle modification.

This is a retrospective study that involved 118 patients (55 women and 63 men) with MetSyn who were under the supervision of a general practitioner (GP) in routine care between 2024 and 2025 in the outpatient clinic. The selected group of MetS patients was motivated to change their diet due to health problems. Out of this group, sixty two patients were supported by trizepatide as a novel drug recommended recently in Poland for patients suffering from obesity. Patients took 2.5 mg of tirzepatide for the first 4 weeks, then 5 mg for the next 4 weeks and finally 7 mg until the end of observation. Meetings with the GP were held monthly to discuss both the nutritional situation and changes in drug dosage—in accordance with the guidelines [12]. After one year of following the GP’s recommendations, the patients’ quality of life (QoL) and consumption frequency of dietary products were assessed. Education on diet modification took place during subsequent “face-to-face” visits in the outpatient clinic and was conducted monthly throughout the year. The GP provided education on healthy eating, highlighting the energy density of foods from various food groups, including dairy products, meat and processed meats, and snacks. The need to limit alcohol consumption and increase physical activity was emphasized. The entire program was supported by dedicated motivational training and based on trust and the patient–doctor relationship built over years of care at the clinic.

The inclusion criteria for the MetS population were based on the 2022 diagnostic consensus of several Polish societies [3] and included: the presence of central obesity (waist circumference ≥ 88 cm for patients or BMI ≥ 30 kg/m^2^) and two and more of the following: a. blood pressure (BP) ≥ 130/85 mmHg or treatment for hypertension; b. non-HDL cholesterol ≥ 130 mg/dL or lipid-lowering treatment; c. fasting plasma glucose (Glc) ≥ 100 mg/dL (5.6 mmol/L) or ≥140 mg/dL after a glucose tolerance test (GTT) or glycated hemoglobin (HbA1c) ≥ 5.7% or hypoglycemic treatment. Exclusion criteria for the study population were based on: cancer diagnosis, autoimmune or chronic inflammatory diseases affecting metabolic parameters, use of drugs affecting lipid or glucose metabolism, pregnancy or lactation, and alcohol or drug abuse.

### 2.2. Biochemical Parameters, Blood Pressure and Indices Used in Adipose Tissue Assessment

Lipid profile and glucose were assessed by routine enzymatic–colorimetric tests (Roche/Hitachi Cobas C system, Switzerland) in accordance with good laboratory practices. The obtained results were interpreted according to the National Health and Nutrition Examination Survey [13] and the American Diabetes Federation [14], respectively. BP was measured with the use of a digital electronic tensiometer (Omron Corp., Kyoto, Japan) following the guidelines of the European Society of Hypertension [15]. Anthropometric measurements, including body weight and body height, were performed using a standardized medical scale (In Body 770, model: BPM040S12FXX, InBody Co., Ltd., Seoul, Republic of Korea, MFG Date 29 May 2018). Waist and hip circumferences were measured with a flexible tape to the nearest 0.1 cm. The following indices were calculated: Body Mass Index (BMI), WHtR (waist-to-height ratio), A Body Shape Index (ABSI), Visceral Adiposity Index (VAI), Lipid Accumulation Product (LAP), and Body Roundness Index (BRI).

BMI = Weight [kg]/Height^2^ [m]

WHtR = WC [cm]/Height [cm]

ABSI = WC/BMI^2/3^ × Height^1/2^

VAI for women = [WC/36.58 + (1.89 × BMI)] × (TG [mmol/L]/0.81) × (1.52/HDL [mmol/L])

VAI for men = [WC/39.68 + (1.88 × BMI)] × (TG [mmol/L]/1.03) × (1.31/HDL [mmol/L])

LAP for women = (WC [cm] − 58) × (TG [mmol/L])

LAP for men = (WC [cm] − 65) × (TG [mmol/L])

BRI = 364.2 − 365.5 × √(1 − [waist circumference [cm]/2π]^2^/[0.5  ×  height cm]^2^)

### 2.3. Nutritional Assessment

The KomPAN^®^ questionnaire was used to assess diet in the study group of MetSyn patients. Selected products from 24 established food categories were grouped as more (e.g., plant-based) and less healthy (e.g., red meat) to calculate: the Non-Healthy Diet Index-14 (nHDI-14) (scores ranged between 0 and 28 points) and the pro-Healthy-Diet-Index-10 (pHDI-10) (scores ranged between 0 and 20 points) with further standardization to a 0–100-point scale for the diet quality score calculation (Table 1) [16]. The MetSyn patients indicated the frequency of consumption for each of the food categories within the following ranges: never (0.0), 1–3 times a month (0.06), once a week (0.14), several times a week (0.5), once a day (1.0), and several times a day (2.0) [16].

### 2.4. Quality of Life Assessment

WHO Quality of Life-BREF (WHOQOL-BREF) was used to assess the QoL of MetSyn patients after one year of diet modification. The questionnaire comprises 26 items which assess several domains: physical health (activities of daily living, dependence on medicinal substances and medical aids, energy and fatigue, mobility, pain and discomfort, sleep and rest, and work capacity), psychological health (body image and appearance, negative feelings, positive feelings, self-esteem, spirituality/religion/personal beliefs, thinking, learning, memory and concentration), social relationships (personal relationships, social support, and sexual activity) and environment (financial resources; freedom; physical safety and security; health and social care—accessibility and quality; home environment; opportunities for acquiring new information and skills; participation in and opportunities for recreation/leisure activities; physical environment; and transport). The mean score of items within each domain was used to calculate the domain score [17].

### 2.5. Statistical Approach

Data normality was assessed using the Shapiro–Wilk test. Normality was not simultaneously observed for any single parameter across all analyzed groups. Descriptive statistics are presented as medians with minimum and maximum values. Changes within each study group (before and after diet modification supported with tirzepatide) were evaluated using the Wilcoxon signed-rank test for non-parametric data. To address the baseline clinical differences between groups and to strictly compare the effectiveness of tirzepatide versus lifestyle intervention alone, Analysis of Covariance (ANCOVA) was employed. In these models, the outcome was the dependent variable, the study group was the fixed factor, and the respective baseline value was included as a covariate. The magnitude of the differences was assessed using Cohen’s d effect size (interpreted as: <0.2 negligible, 0.2–0.5 small, 0.5–0.8 medium, and >0.8 large). For categorical success rates (e.g., ≥5% weight loss), Fisher’s exact test was applied. Comparisons between groups based on the data obtained from the KOMPAN and WHOQOL-BREF questionnaires were conducted using the Mann–Whitney U test. Due to the high number of parallel endpoints in laboratory, dietary, and quality of life analyses, Benjamini–Hochberg False Discovery Rate (FDR) correction was applied to all *p*-values to control for multiplicity. A *p*-value of <0.05 after adjustment was considered statistically significant. The statistical analyses were computed in Statistica version 13, 2017 (TIBCO Software Inc., 3307 Hillview Avenue, Palo Alto, CA 94304, USA).

## 3. Results

The analyzed real-life data of MetSyn patients (median age (min, max): tirzepatide + diet group—46 (24, 78); diet-only group—55 (19, 84); *p* = 0.231) was the result of from routine cooperation with their GP and concerned people who were willing to improve their diet and health condition. During the one-year follow-up period, patients from both groups (the group changing their diet towards health-promoting activities only and the group modifying their diet and supporting this with medication) treated in the GP practice showed health improvements. No severe adverse events or treatment interruptions were reported and no patients required a dose reduction. Patients from both groups significantly reduced their body weight and WC and thus significantly reduced the values of indices assessing nutritional status, visceral adiposity distribution and metabolic risk such as BMI, WHtR, ABSI, LAP and BRI. Additionally, both groups showed a significant increase in LDL cholesterol and TG values. The group following dietary modification with tirzepatide experienced a significant decrease in Glc levels (Table 2). The BP decrease was close to statistical significance in both groups.

The energy value and energy density of food did not differ between groups after 1 year of modification. The supply of main nutrients was at a similar level in both groups, and the intensity of beneficial nutritional features (pHDI-10) was low (Table 3).

Taking into account the detailed percentage of energy that was derived from the analyzed food categories, a significant difference was noted in red meat dishes between the groups, indicating a significantly higher share of these products in the diets of patients following a diet exclusively. This is consistent with the diet analysis and higher total fat intake in these individuals (Table 4).

**Figure 1:**
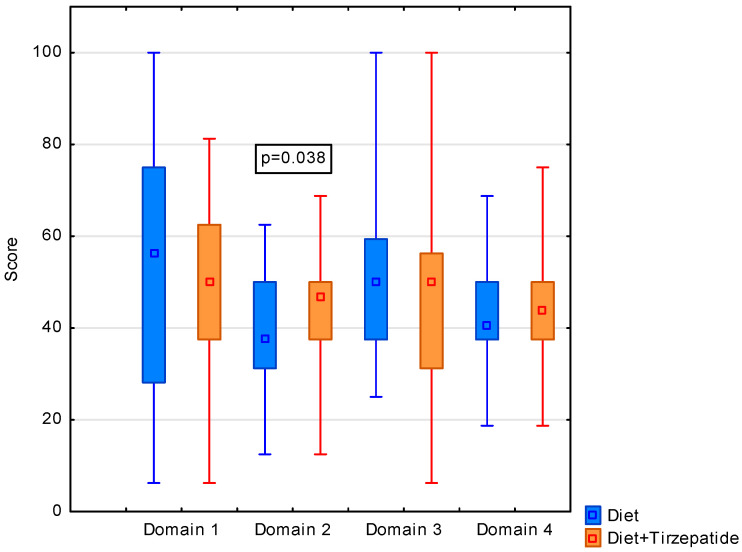
Association between quality of life domains (a. physical health, b. psychological, c. social relationship, and d. environment) and diet modification supported by trizepatide in MetSyn patients (*n* = 118).

We observed differences in QoL between groups in the area of domain 2 (mental health), indicating greater benefits in terms of: self-esteem, mood, cognitive abilities in patients using the drug together with diet modification (median (min, max): diet + trizepatide group—46.9 (2.5, 68.8) vs. diet group—37.5 (12.5, 62.5); *p* = 0.038). Patients from the group with higher values were characterized by a moderate quality of life (46–65 points) (Figure 1). In general, total calculated QoL fluctuated around 50 points in both groups, indicating an average QoL.

## 4. Discussion

Real-life data indicated health benefits for patients with MetSyn after one year of cooperation with a GP in the field of weight loss, both in the case of using the drug together with dietary changes and in the case of only dietary modifications resulting from regular consultations, education and patient motivation. Significant positive changes in visceral adiposity distribution were visible in both groups, with significant improvement in glucose levels when tirzepatide was used. High intake of fat from red meat dishes negatively influenced the lipid profile observed in both groups.

The observed changes in lipid profile in the group with drug administration were not expected, especially since it was indicated that tirzepatide can enhance adipocyte function as it is a modulator of lipid storage in adipocytes, leading to nutrient storage in the fed state and appropriate nutrient release in the fasted state [8]. It was also highlighted by Galindo et al. [8] that in the postprandial state, insulin signaling activates LPL, a key mediator of the intake of triglyceride-derived fatty acids into cells. Of course, the changes visible in the lipid profile are directly related to the patients’ diet. From our previous research, we know that structured motivational training combined with nutritional education can bring lasting results observed in annual follow-up [9]. Unfortunately, with such a busy schedule, not every GP is able to support patients with obesity through long-term motivational training during visits on a regular basis. Perhaps GPs could leverage digital channels to provide dedicated materials accessible at any time of day. Research shows that even people over 65 can successfully develop digital skills, which reduces the potential exclusion of older people in such education [18]. The current study clearly shows that it is worth working one-to-one with patients as this approach is very effective in body weight reduction.

It should be emphasized that higher consumption of ultra-processed food (UPF) by people with obesity is associated not only with a lower diet nutritional value but also with the development of unusual eating behaviors, symptoms of binge eating or bulimia, and emotional eating [19]. Finally, when considering the fat component of a dish, we have to think about the fatty acid profile. A study by Poosri et al. [20] suggests that modulating dietary fat intake can be a potential strategy for mitigating obesity-related inflammation and leptin resistance, highlighting the need for targeted nutritional interventions in this group of people. Widmer et al. [21] found that replacing typical high-carbohydrate snacks with tree nuts results in more favorable dietary, plasma, and adipose tissue fatty acid profiles that could aid in preventing the development of MetSyn. This study allows for a simplified look at the structure of the consumption of specific food groups, which directly translates into the biochemical parameters and body composition of the patient.

In the current study, several novel indices with the potential to predict MetSyn were assessed. A significant decrease in LAP and ABSI indicates a decrease in abdominal fat accumulation. Analysis of NHANES data indicated that higher LAP was associated with increased all-cause (22%) and CVD-specific (14%) mortality, suggesting its potential as a mortality predictor [22]. It is worth mentioning that in Nord-Trøndelag Health Study 2, ABSI and WHtR were more strongly associated with cardiovascular mortality than BMI, WC, or WHR over a mean 17.7-year follow-up [23]. On the other hand, ABSI had lower discriminatory ability for MetS (AUC = 0.762) compared to BRI (AUC = 0.714) [24]. High ABSI has also been linked to sarcopenia and reduced hand grip strength, with weak correlations to visceral fat area on computed tomography [25,26], suggesting caution when interpreting ABSI in the context of abdominal obesity-related metabolic disorders. Interestingly, ABSI may also be influenced by modifiable lifestyle factors, such as sleep duration, as participants sleeping >9 h had a 41.7% higher ABSI, while those sleeping <6 h had a 40.1% lower ABSI [27]. All these factors are extremely important as they constitute determinants of the diet and thus influence the energy and nutritional value of the diet. At the same time, they also affect body weight and nutritional status. In the current study, we were unable to demonstrate the superiority of any of the methods used to support weight loss. In the recently published meta-analysis [28], it was indicated that with the use of tirzepatide we should expect more than 10% body weight reduction, normoglycemia restoration, remission of type 2 diabetes and reduction in hospitalization due to heart failure. Moreover, tirzepatide was effective in the remission of obstructive sleep apnea syndrome and metabolic dysfunction-associated steatohepatitis. Chinese research confirmed that early weight loss with tirzepatide improved cardiometabolic risk factors, suggesting that an early response may be associated with enhanced long-term metabolic health outcomes [29]. This evidence highlights the promising role of this drug in the treatment of patients with MetSyn. On the other hand, the positive impact of education on weight loss continues to emphasize that diet is not only a “background” in the introduction of weight-loss drugs but an integral part of pharmacological treatment.

In the current study patients reported better QoL in the tirzepatide group which is in line with data published by Schmidt et al. [30]. In their meta-analysis, tirzepatide (10 and 15 mg once weekly) significantly improved patient-reported physical function in adults with overweight or obesity, suggesting that tirzepatide enhances perceived physical capacity and QoL. As highlighted by Li et al. [31], tirzepatide may improve physical function through both weight loss-dependent and -independent mechanisms, especially in those with lower baseline physical function. It was shown that diet-related QoL significantly increased with tirzepatide treatment, indicating it as an independent factor of patient satisfaction [32]. Importantly, a study by Gibble et al. [33] indicated that continued tirzepatide treatment was associated with maintaining health-related quality of life (HRQoL) improvement, while treatment withdrawal resulted in the worsening of HRQoL. The improvement in physical function certainly translated into the improvement in mental health observed in our studies.

The strengths of this study are the real-life data analysis that may convince professionals that personalized motivational training is effective. The real-world findings published by Augusto et al. [34] reinforce the potential role of tirzepatide in improving clinical outcomes and QoL for patients with heart failure without diabetes. The limitations of the study include its retrospective nature, sample size, and data obtained from a single center. It would be of interest to analyze the data from different GPs across Poland and other EU countries. Analysis of the nutritional status of patients would enable objective measurement and reference to the obtained indicator values. Finally, it is worth mentioning that many already published articles were funded by the drug manufacturer of tirzepatide, raising concerns about potential conflicts of interest, which was also highlighted by Franco et al. [35].

## 5. Conclusions

Support in primary care by a physician was successful from a long-term perspective in the group using tirzepatide in combination with dietary modification as well as in the group based on dietary modification only. The data do not indicate a significant advantage of any one approach for patients, prioritizing an individualized approach to treatment.

## Figures and Tables

**Table 1:** Description of analyzed food categories.

Food Categories	Types of Foods in the Category
White bread/Bakery products	Buns, bread, rice cakes
Wholegrain bread	Buckwheat bread, starch bread, buckwheat starch bread
Cereal products (groats/pasta)	Buckwheat, white rice, gluten-free pasta
Milk	2% Milk, 3.2% milk, 0.5% milk
Fermented milk beverages	Natural yogurt, kefir, buttermilk
Cottage cheese/Curd cheese	Low-fat, reduced-fat, full-fat
Hard cheese/Processed cheese	Gouda, edam, processed
White meat dishes	Roasted chicken, chicken with vegetables, chicken cutlets
Red meat dishes	Roast beef, beef escalopes, roast pork, pork loin
Processed meat (cold cuts)	Country ham, Sopot loin, sausage
Fish	Fresh cod, fresh herring, fresh mackerel, pollock
Eggs	Chicken eggs
Pulses (legumes)	White beans, dried peas, red lentils
Vegetables	Carrots, tomatoes, cauliflower, beets
Fruits	Apple, banana, strawberries, orange
Fast food	Risotto
Savory snacks	Corn puffs
Sweets/Confectionery	Dark chocolate, milk chocolate, shortbread cookies
Fried dishes	Breaded pork chop
Butter	Butter
Vegetable oils/Margarines	Rapeseed oil, “Rama” margarine, lard
Sweetened beverages	Coca Cola, etc., fruit drinks
Fruit juices	Apple juice, orange juice, grapefruit juice
Energy drinks	Fruit-flavored carbonated drinks

**Table 2:** Changes in clinical characteristics of MetSyn patients during diet modification supported by trizepatide (*n* = 118).

Analyzed Parameter	Diet Modification + Tirzepatide (*n* = 62) Median (Min, Max)		*p*-Value	Diet Modification (*n* = 56) Median (Min, Max)		*p*-Value	*p*-Value Between Groups After 1 Year (FDR)	Effect Size (Cohen’s d) (95% CI)
	Before	After		Before	After			
Body weight [kg]	107 (73, 161)	102 (76, 152)	0.0003	105 (73, 143)	101.5 (68, 133)	0.000006	0.396 (0.634)	−0.07 (−0.43, 0.3)
WC [cm]	100 (78, 140)	94 (66, 150)	0.0006	99.5 (79, 121)	94 (69, 120)	0.0005	0.963 (0.965)	0.13 (−0.23, 0.5)
BMI [kg/m^2^]	38.84 (25.26, 54.36)	36.27 (28.07, 55.83)	0.0004	37.4 (24.54, 55.86)	35.25 (22.72, 51.56)	0.000002	0.488 (0.651)	−0.07 (−0.43, 0.3)
WHtR	0.6 (0.46, 0.9)	0.57 (0.37, 0.9)	0.0006	0.6 (0.46, 0.72)	0.56 (0.42, 0.72)	0.0005	0.892 (0.965)	0.13 (−0.23, 0.49)
ABSI	0.07 (0.05, 0.11)	0.07 (0.05, 0.1)	0.0007	0.07 (0.05, 0.1)	0.07 (0.05, 0.09)	0.0009	0.327 (0.619)	0.22 (−0.15, 0.58)
VAI	2.19 (1.04, 7.66)	2.27 (0.95, 6.57)	0.706	1.74 (0.73, 5.31)	1.87 (0.75, 6.7)	0.240	0.231 (0.619)	0.06 (−0.31, 0.42)
LAP	86.61 (43.12, 203.39)	78.06 (18.51, 249.49)	0.004	82.63 (34.13, 224.74)	74.65 (17.23, 204)	0.007	0.666 (0.82)	0.07 (−0.3, 0.43)
BRI	5.51 (2.64, 13.93)	4.68 (1.28, 13.96)	0.0007	5.34 (2.58, 8.55)	4.5 (1.91, 8.32)	0.0006	0.965 (0.965)	0.14 (−0.23, 0.5)
TC [mg/dL]	180.7 (150.6, 220)	177.7 (133.9, 254.9)	0.111	180.7 (130.6, 278.3)	181.45 (145.3, 239.2)	0.786	0.446 (0.648)	0.18 (−0.19, 0.54)
LDL-C [mg/dL]	112.3 (11.7, 166.3)	119.05 (89.4, 165.3)	0.043	111.05 (89.4, 167.8)	116.9 (98.4, 187.4)	0.043	0.151 (0.612)	−0.13 (−0.5, 0.23)
HDL-C [mg/dL]	53.2 (34.5, 88.6)	51.55 (33.5, 90.3)	0.275	67.35 (30.9, 99.4)	55.4 (37.8, 90.5)	0.096	0.261 (0.619)	−0.08 (−0.44, 0.29)
TG [mg/dL]	189.55 (165.8, 320.5)	210.6 (132.4, 345.6)	0.005	188.5 (157.8, 320.6)	190.8 (165.1, 347.2)	0.022	0.153 (0.612)	−0.1 (−0.46, 0.27)
Glc [mg/dL]	185.85 (90.2, 211.8)	99.75 (74.3, 301.4)	0.000004	131.65 (70.5, 220.7)	109.65 (67.8, 209.7)	0.413	0.348 (0.619)	0.45 (0.08, 0.82)
hsCRP [mg/L]	3.7 (1.1, 9.9)	3.2 (1.1, 11.2)	0.960	3.85 (1.2, 10.9)	4.35 (1.1, 10.9)	0.265	0.34 (0.619)	0.07 (−0.3, 0.43)
SBP [mmHg]	165.6 (115.4, 190.6)	167.7 (110.9, 199.7)	0.396	167.1 (118.4, 201.3)	160.8 (122.4, 199.7)	0.097	0.07 (0.612)	−0.31 (−0.67, 0.06)
DBP [mmHg]	76.7 (33.8, 98.7)	67.5 (44.5, 94.5)	0.072	75.4 (44.3, 96.5)	65.4 (40.5, 89.7)	0.062	0.15 (0.612)	−0.07 (−0.43, 0.3)

BMI—Body Mass Index; hsCRP—high-sensitive C-reactive protein; Glc—glucose; TG—triglycerides; HDL-C—high-density lipoprotein cholesterol; LDL-C—low-density lipoprotein cholesterol; TC—total cholesterol; WHtR—waist-to-height ratio; VAI—Visceral Adiposity Index; LAP—lipid accumulation product; ABSI—A Body Shape Index.

**Table 3:** Energy and nutritional value of diet in MetSyn patients (*n* = 118).

Analyzed Parameter	Diet Modification + Tirzepatide (*n* = 620) Median (Min, Max)	Diet Modification (*n* = 56) Median (Min, Max)	*p*-Value (FDR)
Energy [kcal]	2142.19 (826.73, 3947.72)	2210.11 (871.37, 3508.84)	0.642 (0.693)
Energy density [kcal/100 g]	144.77 (86.43, 214.09)	143.11 (79.63, 194.38)	0.677 (0.693)
Protein [g]	123.59 (36.91, 238.78)	122.03 (41.93, 229.79)	0.693 (0.693)
Carbohydrate [g]	181.18 (54.84, 337.25)	184.01 (83.08, 334.01)	0.685 (0.693)
Fat [g]	68.96 (25.84, 116.02)	70.95 (21.24, 124.43)	0.693 (0.693)
nHDI-14	22.5 (8.46, 44.34)	22.68 (8.46, 44.94)	0.558 (0.693)
pHDI-10	24.9 (8, 56.4)	26 (7.5, 50.7)	0.512 (0.693)

nHDI-14—non-Healthy Diet Index-14; pHDI-10—pro-Healthy-Diet-Index-10.

**Table 4:** Percent of total energy intake provided by food category in MetSyn patients (*n* = 118).

Food Categories	Diet Modification + Tirzepatide (*n* = 62) Median (Min, Max)	Diet Modification (*n* = 56) Median (Min, Max)	*p*-Value (FDR)
	% Energy	% Energy	
White bread/Bakery products	0.83 (0, 10.91)	1.8 (0, 11.75)	0.391 (0.847)
Wholegrain bread	0.71 (0, 11.84)	0.93 (0, 12.3)	0.912 (0.912)
Cereal products (groats/pasta)	1.33 (0, 21.9)	2.7 (0, 23.68)	0.500 (0.847)
Milk	0.45 (0, 1.94)	0.43 (0, 10.61)	0.616 (0.847)
Fermented milk beverages	1.4 (0, 13.72)	1.9 (0, 14.53)	0.874 (0.912)
Cottage cheese/Curd cheese	3.82 (0, 29.54)	1.58 (0, 17.82)	0.066 (0.528)
Hard cheese/Processed cheese	1.76 (0, 13.64)	0.73 (0, 8.23)	0.066 (0.528)
White meat dishes	3.91 (0, 27.36)	2.08 (0, 30.41)	0.258 (0.847)
Red meat dishes	1.59 (0, 33.34)	5.08 (0, 33.1)	0.032 (0.528)
Processed meat (cold cuts)	0.42 (0, 5.76)	0.41 (0, 4.47)	0.337 (0.847)
Fish	5.33 (0, 23.01)	1.68 (0, 22.74)	0.103 (0.618)
Eggs	0.98 (0, 8.74)	0.72 (0, 8.92)	0.571 (0.847)
Pulses (legumes)	3.41 (0, 17.87)	2.68 (0, 19.44)	0.582 (0.618)
Vegetables	0.23 (0, 4.86)	0.49 (0, 3.28)	0.419 (0.847)
Fruits	0.57 (0, 9.42)	0.83 (0, 7.29)	0.524 (0.847)
Fast food	11.18 (0, 51.18)	7.68 (0, 43.62)	0.717 (0.906)
Savory snacks	3.24 (0, 19.1)	1.83 (0, 17.87)	0.164 (0.787)
Sweets/Confectionery	3.84 (0, 27.77)	5.15 (0, 27.08)	0.635 (0.847)
Fried dishes	6.81 (0, 42)	7.2 (0, 50.31)	0.861 (0.912)
Butter	0.46 (0, 8.22)	0.35 (0, 8.48)	0.467 (0.847)
Vegetable oils/Margarines	0.44 (0, 7.85)	0.34 (0, 8.1)	0.467 (0.847)
Sweetened beverages	0.93 (0, 19.62)	2.04 (0, 14.42)	0.209 (0.836)
Fruit juices	2.47 (0, 23.65)	2.19 (0, 13.95)	0.912 (0.912)
Energy drinks	1.91 (0, 13.57)	1.54 (0, 20.4)	0.908 (0.912)

## Data Availability

The original contributions presented in this study are included in the article. Further inquiries can be directed to the corresponding author.

## Abbreviations

The following abbreviations are used in this manuscript:

MetSyn	Metabolic Syndrome
GP	General Practitioner
BMI	Body Mass Index
WHtR	Waist-to-Height Ratio
BRI	Body Roundness Index
ABSI	A Body Shape Index
LAP	Lipid Accumulation Product
VAI	Visceral Adiposity Index
QoL	Quality of Life
BP	Blood Pressure
FFQ	Frequency of Food Consumption
GTT	Glucose Tolerance Test
HbA1c	Glycated Hemoglobin
WC	Waist Circumference
TG	Triglyceride
nHDI-14	Non-Healthy Diet Index-14
pHDI-10	Pro-Healthy-Diet-Index-10
